# Pain and pain mechanisms in patients with inflammatory arthritis: A Danish nationwide cross-sectional DANBIO registry survey

**DOI:** 10.1371/journal.pone.0180014

**Published:** 2017-07-07

**Authors:** S. Rifbjerg-Madsen, A. W. Christensen, R. Christensen, M. L. Hetland, H. Bliddal, L. E. Kristensen, B. Danneskiold-Samsøe, K. Amris

**Affiliations:** 1 The Parker Institute, Copenhagen University Hospital, Bispebjerg and Frederiksberg, Frederiksberg, Denmark; 2 DANBIO, Center for Rheumatology and Spine Diseases, Rigshospitalet, Glostrup, Denmark; 3 Department of Clinical Medicine, Faculty of Health and Medical Sciences, University of Copenhagen, Copenhagen, Denmark; 4 Department of Rheumatology, Copenhagen University Hospital, Bispebjerg and Frederiksberg, Frederiksberg, Denmark; University of Twente, NETHERLANDS

## Abstract

**Background:**

Central pain mechanisms may be prominent in subsets of patients with rheumatoid arthritis (RA), psoriatic arthritis (PsA) and other spondyloarthritis (SpA). The painDETECT questionnaire (PDQ) identifies neuropathic pain features, which may act as a proxy for centrally mediated pain.

The objectives were to quantify and characterize pain phenotypes (non-neuropathic vs. neuropathic features) among Danish arthritis patients using the PDQ, and to assess the association with on-going inflammation.

**Methods:**

The PDQ was included onto the DANBIO touch screens at 22 departments of Rheumatology in Denmark for six months. Clinical data and patient reported outcomes were obtained from DANBIO. A PDQ-score >18 indicated neuropathic pain features, 13–18 unclear pain mechanism and <13 non-neuropathic pain.

**Results:**

Pain data (visual analogue scale, VAS) was available for 15,978 patients. 7,054 patients completed the PDQ (RA: 3,826, PsA: 1,180, SpA: 1,093). 52% of all patients and 63% of PDQ-completers had VAS pain score ≥ 30 mm. The distribution of the PDQ classification-groups (<13/ 13-18/ >18) were; RA: 56%/24%/20%. PsA: 45%/ 27%/ 28%. SpA: 55% / 24%/ 21%. More patients with PsA had PDQ score >18 compared to RA and SpA (p<0.001). For PDQ > 18 significantly higher scores were found for all patient reported outcomes and disease activity scores. No clinical difference in CRP or swollen joint count was found. Logistic regression showed increased odds for having VAS pain ≥39 mm (the median) for a PDQ-score >18 compared to <13 (OR = 10.4; 95%CI 8.6–12.5).

**Conclusions:**

More than 50% of the Danish arthritis patients reported clinically significant pain. More than 20% of the PDQ-completers had indication of neuropathic pain features, which was related to a high pain-level. PDQ-score was associated with DAS28-CRP and VAS pain but not with indicators of peripheral inflammation (CRP and SJC). Thus, pain classification by PDQ may assist in mechanism-based pain treatment.

## Introduction

Pain in rheumatoid arthritis (RA), psoriatic arthritis (PsA) and other spondyloarthritis (SpA) is traditionally considered to be of peripheral nociceptive origin, i.e. pain elicited by activation of afferent sensory nerve fibers (C-fibers) in the inflamed synovium [1]. Increased responsiveness of peripheral and central nociceptive neurons, i.e. pain hypersensitivity, is a normal response (neuroplasticity) in the presence of inflammation. However, pain hypersensitivity may persist after the inflammation has ceased and thus become a manifestation of maladaptive pathological changes in the central nervous system leading to chronic pain [2].

Chronic pain has been most thoroughly investigated in RA where pain is reported to persist despite regression of inflammatory signs [3] and emerging data suggest a role of augmented central pain processing, including central sensitization and dysfunction of descending pain modulating systems in subsets of patients [4]. Fibromyalgia (FM), by many considered the prototypical central pain syndrome, is more common in RA than in the general population with reported prevalence estimates of up to 20% [5]. A co-diagnosis of FM in RA is associated with poorer outcome of anti-inflammatory treatment when evaluated by pain ratings and composite disease activity scores [5;6]. However, it is likely that not only individuals with RA and concomitant FM have abnormal central pain processing. There is probably a continuum of pain hypersensitivity among patients with RA that may influence patient-reported outcomes and disease activity evaluations [6]. Subserving pain mechanisms in PsA and SpA are less well examined and studies have mainly focused on co-occurrence of FM. The reported prevalence of FM ranges from 4% to 15% for SpA and 17% to 53% for PsA depending on screening tool and gender [7–11].

Treatment of inflammatory arthritis is based on disease activity measures that are fully or partially composed of subjective indicators related to pain perception. Pain hypersensitivity may lead to continuous high reports of for example tender joints, pain and poor global health and thus an overestimation of inflammatory activity. Identification of possible underlying pain mechanisms may, therefore, be of great importance and assist clinical decision making. The painDETECT questionnaire (PDQ) is a patient administered screening questionnaire originally developed to identify neuropathic pain [12]. Based on pain phenotypic similarities, the PDQ has been used to assess neuropathic pain features as a proxy of central pain mechanisms in patients with osteoarthritis and fibromyalgia [13–18]. Recently, the PDQ has also been introduced in studies of smaller samples of patients with RA [19;20] and SpA [21] reporting neuropathic pain features in subsets of patients.

The purpose of this study was to report the prevalence of pain phenotypes (non-neuropathic vs. neuropathic pain features) as assessed by the PDQ in the Danish arthritis population (RA, PsA and other SpA) and to investigate the association between pain phenotype and inflammation. Furthermore, the study aimed to describe differences in patient characteristics across PDQ-classification groups.

## Patients and methods

### Study design and setting

The study was designed as a descriptive, cross sectional survey including patients registered in DANBIO, the Danish nationwide rheumatologic registry (Protocol; http://parkerinst.dk/research). The DANBIO-registry is a clinical quality assurance registry which was initiated in year 2000. It is based on Danish rheumatologists registering and continuously reporting data on their patients diagnosed with inflammatory arthritis when seen in daily care. It covers >90% of adults treated with biologics due to rheumatic disease and data on patients treated with disease-modifying antirheumatic drugs, DMARDs, are also collected [22–24].

Following local approval of the project at the departments, an electronic version of the PDQ was implemented nationally on the DANBIO touch screens in the waiting room at 22 of 24 departments of Rheumatology in Denmark for a period of six months (1.Dec 2013-1.June 2014). All patients registered as having any form of RA, PsA, other SpA, unspecific arthritis (UA) or ‘no diagnosis’ were eligible and invited to participate in the survey. Patients were asked to accept participation, decline or indicate ‘no pain 4 weeks prior to the survey’ on the touch screens. Patients choosing the two latter categories were excluded from filling in the PDQ, while clinical data from DANBIO were obtainable from all patients visiting the touch screens in the study period.

The primary focus was on the diagnostic groups: RA, PsA and other SpA. All patients were included in the overall pain analysis, while only patients with a complete PDQ response were included in the PDQ subanalyses. The first complete questionnaire and corresponding clinical data from the same date were extracted for analyses (CRP +/- 14 days).

Patient consent was obtained on the touch screen prior to the redirection to the PDQ. According to Danish legislation, surveys do not require approval by Ethics Committees and registrations and publications of data from clinical registries do not require patient consent or approval by Ethics Committees. Approval was obtained from the Danish Data Protection Agency.

### Variables and outcome measures

The PDQ is a symptom-based assessment tool originally developed to assist identification of neuropathic pain in patient with low back pain [12]. It is composed of items reflecting pain intensity (three numeric rating scales), pain course pattern, a pain drawing intended for indication of pain radiation, and seven questions describing somatosensory signs and symptoms considered characteristic for neuropathic pain rated on a six-category Likert scale (from never to very strongly). A total score ranging from -1 to 38 is calculated based on the patient’s answers. Pain intensity ratings are not included in the total score; selection of pain course pattern contributes to the total score with a value ranging from -1 to 1; the absence/presence of radiating pain with a value of 0 or 2; and the presence and severity of evaluated somatosensory signs and symptoms with a value ranging from 0 to 35. For diagnostic purposes, a validated algorithm is used to classify pain into three groups: a score >18 indicate that presence of a predominant neuropathic pain component is likely, a score <13 indicated that it is not, while a score of 13–18 is considered indecisive; i.e., a neuropathic pain component cannot be ruled out. [12]. The questionnaire is reported to have a sensitivity of 84% and likewise a specificity of 84% (electronic version) when applied for pain classification in a mixed chronic pain population [12]. Satisfactory psychometric properties of the PDQ have been demonstrated within osteoarthritis by Morton et al.[25], and within inflammatory arthritis by our group (in review).

Information on demographics and treatment were registered according to the DANBIO registry standard. Before consulting their rheumatologist patients regularly complete commonly used standardized patients reported outcomes (Table 1) on the DANBIO touch screens in the doctor’s waiting room. All patients complete visual analogue scale (VAS) pain, VAS fatigue, and VAS global health, which are 0–100 mm scales, where 100 mm indicates the worst imaginable pain/fatigue/ general health. Depending on their diagnosis patients furthermore complete a disability index. For patients with peripheral joint disease the health assessment questionnaire (HAQ)[26], an index reflecting level of function in daily living is used. For patients with axial joint disease the Bath ankylosing spondylitis function index (BASFI) is used [27]. Finally the Bath ankylosing spondylitis disease activity index (BASDAI) is used to register overall disease activity in this patient group [28;29]. The two latter questionnaires include questions on pain, disability and function related to the axial and large joints. The rheumatologists register corresponding clinical outcomes hence different disease activity scores are available in the DANBIO registry:

DAS28-crp (disease activity score 28 joints–CRP) [30] used for peripheral joint disease:

$$\underline{0.56*\surd \;(TJC28)+0.28*\surd \;(SJC28)+0.36*ln(CRP+1)+0.014*GH+0.96}$$

ASDAS (ankylosing spondylitis disease activity score) [31] used for axial joint disease:

$$\underline{0.12*Back\;Pain+0.06*Duration\;of\;Morning\;Stiffness+0.11*Patient\;Global+0.07*Peripheral\;Pain/Swelling+0.5\;Ln\;(CRP+1)}$$

**Table 1:** Overview of patient reported outcome measures and disease activity scores available in the DANBIO registry.

Variable	Collected in disease area	Unit and range*	Aims to assess	Nature of variable^#^
VAS pain	RA/PsA/SpA	0–100 mm	Pain intensity	Self-reported
VAS fatigue	RA/PsA/SpA	0–100 mm	Fatigue severity	Self-reported
VAS global health	RA/PsA/SpA	0–100 mm	Impact on global health	Self-reported
HAQ	RA/PsA	0–3	Disability	Self-reported
BASMI	PsA/SpA	0–100**	Spinal mobility	Clinical examination
BASFI	PsA/SpA	0–100**	Disability	Self-reported
BASDAI	PsA/SpA	0–100**	Overall disease activity	Self-reported
DAS28-CRP	RA /PsA	0–10	Overall disease activity	Combination
ASDAS	PsA/SpA	0–6.3***	Overall disease activity	Combination

*No unit specified indicates an arbitrary unit

** As used in the DANBIO registry

***Maximum scores on all self-reported items (0–10) and CRP 100.

# The nature can be either: Self-reported, clinical examination or a combination of variables that are self-reported, objective and the result of a clinical examination

BASMI-365 (Bath ankylosing spondylitis metrology index undertaken within the last year) [32]: a standardized clinical examination reporting degree of joint stiffness.

### Statistical analysis

SAS software (version 9.3; SAS Institute Inc., Cary; North Carolina, USA) was used for the statistical analyses. The PROC UNIVARIATE statement was used for visual inspection of the distribution of variables. Potential group differences were tested using the non-parametric Kruskal-Wallis test for continuous variables and Chi-square test for categorical variables. Correcting for multiple testing (65 repeated tests) two-sided p-values < 0.001 (0.05/65) were regarded as statistically significant when comparing potential PDQ classification-group differences. Due to the ‘real life’ character of DANBIO complete data for all variables were not available. Only available data were used for calculations.

To address the possible presence of sensitization in more ways also the swollen to tender joint count ratio (STR) was calculated according to previously published procedures [33] and compared across PDQ classification-groups using the PROC FREQ procedure.

To quantify a potential increased risk for rating higher than or equal to the median VAS-pain associated with the PDQ classification-groups (<13 = low, 13–18 = intermediate, >18 = high) logistic regression analysis was performed. For pragmatic reasons the median VAS for PDQ-completers was chosen as cut off (39 mm). Prior to conducting the logistic regression, an analysis of possible interaction between the PDQ classification-group and diagnosis was performed, which proved insignificant (y = ‘diagnosis’ ‘PDQ classification-group’ ‘diagnosis*PDQ classification-group’ (interaction); p-value for interaction = 0.76, p-value for PDQ ‘classification-group’ <0.001, p-value for ‘diagnosis’ < 0.001). Hence, the ‘PDQ classification-group’ and ‘diagnosis’ were applied as main effects in the unadjusted Model 1. Separate models were run for each pain classification and disease comparison. Based on clinical significance and plausibility the following a priori selected confounders were included in the adjusted Model 1: sex, age, disease duration, CRP, and status of treatment with biologics (yes/no). Accepted level of covariance between independent variables was Spearman’s Rho < 0.3. Backward deletion of variables with a p-value > 0.1 was performed to make a more parsimonious adjusted model (Model 2). Results from the logistic regression analyses are reported as odds ratios with confidence intervals (95%CI).

## Results

### Flow of respondents and sampling process

**Fig 1:**
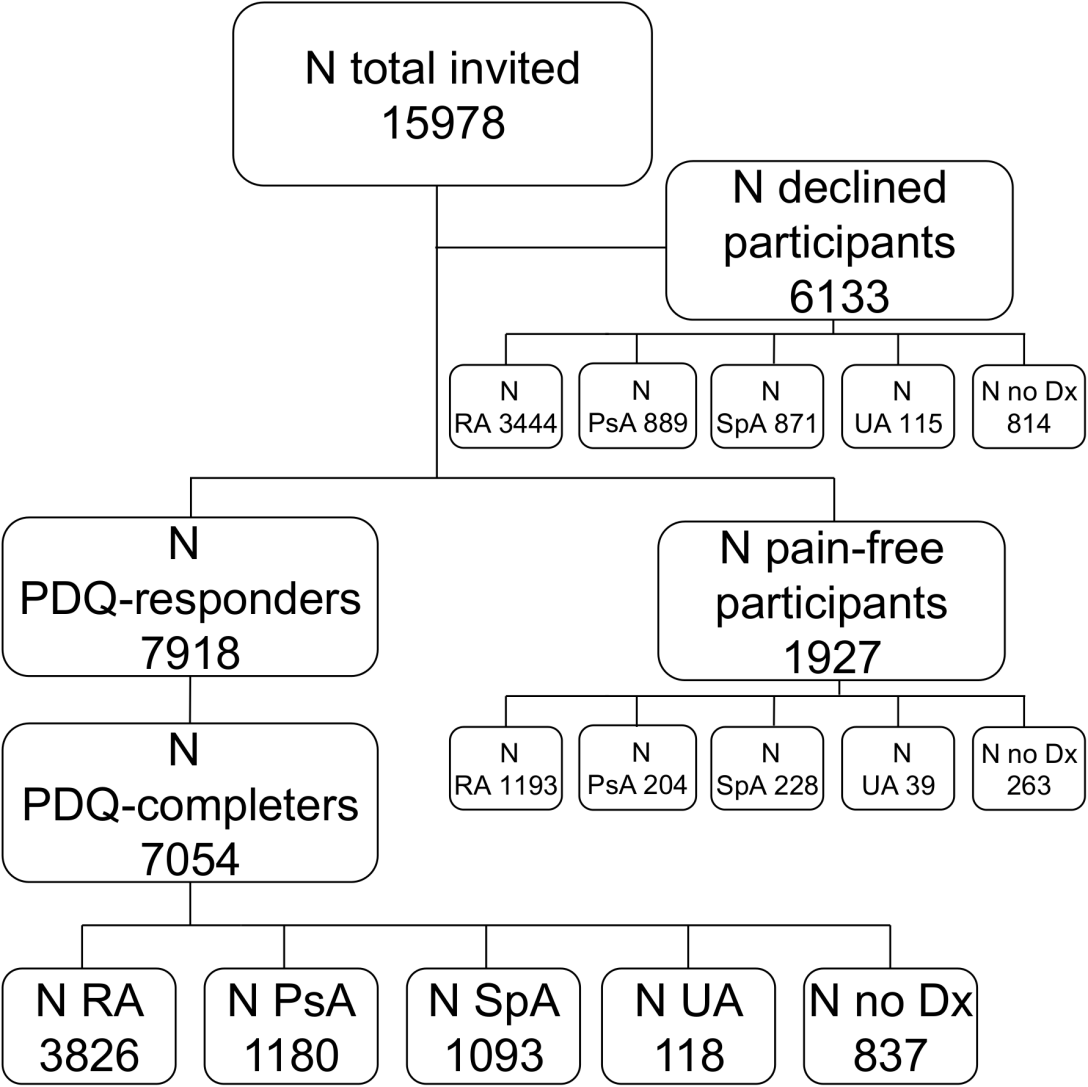
Flowchart of possible participants in the painDETECT survey. RA: rheumatoid arthritis; PsA: psoriatic arthritis; SpA: other Spondyloarthritis; UA unspecified arthritis; Dx: diagnosis.

Fig 1 illustrates the patient flow. A total of 15,978 patients belonging to either of the following diagnostic groups; RA, PsA, other SpA, unspecific arthritis (UA) or ‘no diagnose’ were invited to participate in the study (visited the touch screens in the period). Standard clinical data from DANBIO were available on these patients. Of those 7,918 accepted to fill in the PDQ and 7,054 completed all items in the questionnaire and were assigned a score. A total of 6,133 patients declined participation, while 1,927 registered themselves as being pain-free, thus principally excluding themselves from filling in the PDQ. The median (IQR) level of VAS pain reported as standard in the DANBIO-registry for all invited participants was 31 (14–55) mm, 39 (21–63) mm for PDQ-completers, 30 (14–53) mm for declining participants and 8 (2–20) mm for participants categorizing themselves as pain-free in the introductory touch screen form. Of all patients visiting the touch screen 52% (7,483/14,339) had a VAS pain ≥ 30 mm while the numbers were 63% (3,991/6,380) among PDQ-completers, 51% (2,799/5,471) for declining participants and 15% (262/1,712) for participants reporting to be pain-free.

### PDQ classification across arthritis diagnoses

**Fig 2:**
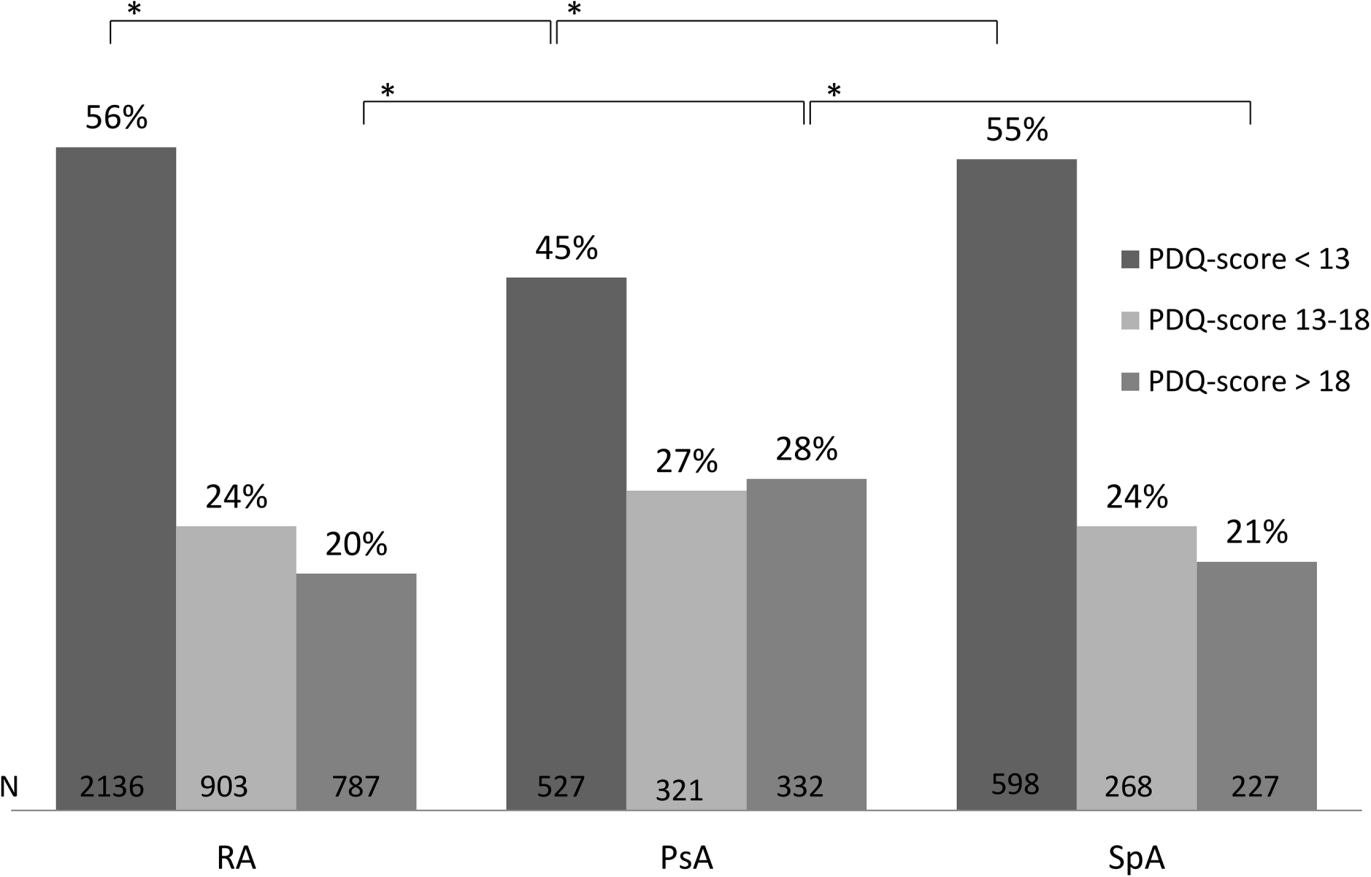
Distribution of painDETECT classification-groups (<13/ 13-18/ >18). * p < 0.001.

The main results of this study, the distribution of the PDQ classification-groups (<13/ 13-18/ >18), are illustrated in Fig 2. More than 20% across all diagnoses had a PDQ score > 18 indicative of primarily neuropathic pain features. A total of 28% of PsA patients had a PDQ scores > 18 which was significantly higher than for patients with RA and SpA (p<0.001). Also statistically significantly fewer PsA patients had a PDQ score < 13 (p<0.001) indicating primarily non-neuropathic pain.

### Participant demographics and clinical characteristics

Patient characteristics stratified by PDQ-score are shown in Table 2. Treatment with DMARDs included: methotrexate (MTX), sulfasalazine, leflunomide, chloroquine and azathioprine. Treatment with Biologics primarily included one of the following agents: infliximab, eternacept, adalimumab, golimumab, certolizumab, abatacept, tocilizumab, or rituximab. MTX in either mono or combination therapy was used in 71% of RA, 58% of PsA and 15% of SpA patients.

**Table 2:** Patient characteristics stratified by painDETECT classification-group.

		n	<13	PDQ score 13–18	>18	P-value
**Demographics**						
Patients, total (n)	RA	3826	2136	903	787	<0.001†
	PsA	1180	527	321	332	<0.001†
	SpA	1093	598	268	227	<0.001†
Female sex n(%)^a^	RA	2766	1455 (68)	674 (75)	637 (81)	<0.001†
	PsA	666	235 (45)	193 (60)	238 (72)	<0.001†
	SpA	436	194(32)	123 (46)	119 (52)	<0.001†
Age (yrs)	RA	3826	61 (52–69)	59 (51–67)	59 (51–66)	<0.001†
	PsA	1180	53 (43–61)	52 (44–60)	52 (42–60)	0.227
	SpA	1093	45 (36–55)	45 (37–55)	43 (36–53)	0.213
Disease duration^b^	RA	3470	8(3–16)	8(3–15)	8(3–15)	0.573
	PsA	1000	7 (3–12)	5 (2–10)	5 (2–10)	0.004
	SpA	953	7 (3–15)	5 (2–10)	4 (2–10)	0.001
**Treatment intensity**						
DMARDS n(%)	RA	3216	1829(86)	749(83)	647(82)	0.035
	PsA	792	349(66)	222(69)	221(67)	0.656
	SpA	237	122(20)	62(23)	53(23)	0.527
DMARDS>1^c^	RA	777	395(18)	197(22)	185(24)	0.005
	PsA	79	27 (5)	25(8)	27(8)	0.150
	SpA	18	12 (2)	3 (1)	3 (1)	0.581
Current bio n(%)	RA	1341	730(34)	328(36)	283(36)	0.439
	PsA	458	212(40)	121(38)	125(38)	0.670
	SpA	646	362 (61)	153(57)	131 (58)	0.566
Bio >1^d^	RA	875	422 (50)	240 (59)	213 (59)	0.002
	PsA	263	88 (36)	72 (51)	103 (61)	<0.001†
	SpA	349	162(40)	93 (49)	94 (59)	0.003
DMARDs previous bio^e^	RA	204	80 (4)	60 (7)	64 (8)	<0.001†
	PsA	59	20 (4)	13(4)	26(8)	0.020
	SpA	11	7 (1)	7(3)	4 (2)	0.302
Prednisolone n(%)	RA	376	188 (9)	87 (10)	101 (13)	0.005
	PsA	28	12 (2)	6 (2)	10 (3)	0.619
	SpA	11	8 (1)	2 (1)	1 (0.5)	0.456
**PROs**						
VAS pain	RA	3508	26 (14–44)	43 (27–61)	61 (43–77)	<0.001†
	PsA	1071	27 (15–46)	49.5 (29–69)	69 (52–81)	<0.001†
	SpA	995	30 (16–53)	53 (36–71)	68.5 (52–82)	<0.001†
VAS fatigue	RA	3508	34 (17–57)	54 (34–72)	72 (54–85)	<0.001†
	PsA	1070	35 (17–57)	63.5 (40–80)	78 (62–90)	<0.001†
	SpA	995	40 (22.5–66)	65 (41.5–81)	77 (60–88)	<0.001†
VAS GH	RA	3522	29 (15–53)	51 (31–68)	70 (52–84)	<0.001†
	PsA	1071	30 (15–57)	58 (34–78)	77 (59–89)	<0.001†
	SpA	994	33.5 (19–56)	63 (39.5–77.5)	75 (59–87)	<0.001†
HAQ	RA	3506	0.5 (0.125–1)	1 (0.5–1.375)	1.375(1–1.875)	<0.001†
	PsA	1046	0.375(0.125–0.75)	0.875(0.5–1.375)	1.375(1–1.875)	<0.001†
BASDAI	PsA	1002	29 (15–46)	53 (36–68)	70 (55–83)	<0.001†
	SpA	923	31.5 (17–48)	53 (35–66)	68 (53–79)	<0.001†
BASFI	PsA	1001	22 (8–38)	42.5 (26–61)	65 (46–81)	<0.001†
	SpA	921	23 (11–43)	38 (21–58)	58 (41–77)	<0.001†
**Disease activity scores**						
VAS doctor	RA	3058	7 (3–14)	10 (4–19)	13 (6.5–26)	<0.001†
	PsA	900	8 (3–15)	10 (5–20)	15 (7–25)	<0.001†
	SpA	609	6 (2–15)	9 (4–16)	12 (5–25)	<0.001†
DAS28-CRP	RA	3046	2.4 (1.9–3.3)	3(2.3–3.9)	3.7 (2.8–4.7)	<0.001†
	PsA	825	2.4 (1.8–3.0)	3.1(2.3–3.9)	3.7 (2.7–4.6)	<0.001†
SJC	RA	3282	0 (0–1)	0 (0–2)	0 (0–2)	<0.001†
	PsA	946	0 (0–0)	0 (0–1)	0 (0–1)	0.007
TJC	RA	3295	0 (0–2)	1 (0–5)	4 (0–9)	<0.001†
	PsA	956	0 (0–2)	1 (0–4)	4 (0–9)	<0.001†
ASDAS	PsA	823	2 (1.4–2.7)	2.6 (2.1–3.55)	3.45 (2.7–3.95)	<0.001†
	SpA	697	2 (1.4–2.8)	2.9 (2.2–3.3)	3.2 (2.6–3.9)	<0.001†
BASMI-365	PsA	299	10 (0–20)	10 (0–30)	20 (10–30)	0.004
	SpA	921	10 (0–40)	20 (10–30)	20 (10–40)	0.010
**Biochemistry**						
CRP mg/l	RA	3231	4 (2–9)	4 (1–9)	4 (2–10)	0.067
	PsA	914	3 (1–6)	3 (1–7)	4 (2–7)	0.446
	SpA	775	3 (1–7)	3 (1–7)	3 (1–8)	0.736
RF n(%)	RA	3091*	1388 (78)	575 (78)	455 (78)	0.968
antiCCP n(%)	RA	1908*	579 (53)	221 (48)	173 (48)	0.122
HLA-B27 n(%)	PsA	128*	36 (60)	21 (58)	13 (41)	0.180
	SpA	367*	167 (82)	61 (71)	45 (58)	0.002

All values are the median (Q1-Q3) unless otherwise specified. Group differences were tested using Kruskal-Wallis test or Chi-square test.

a: Refers to the fraction of women in each subgroup of PDQ classification. b: Time since diagnosis. c: Receives a combination of DMARDs. d: Have received more than one biologic agent. e: Currently treated with DMARDs and previously treated with a biologic agent.

† Remain significant after adjusting for multiple testing.

*Number of patients with available information (pos/neg).

PDQ-score: painDETECT Questionnaire score; DMARDs: disease modifying anti-rheumatic drugs; Bio: biological agent; PROs: patient reported outcomes; VAS: visual analogue scale (mm); GH: global health; DAS28-CRP: disease activity score 28 joints; CRP: C- reactive protein; SJC: swollen joint count; TJC: tender joint count. HAQ: health assessment questionnaire; ASDAS: ankylosing spondylitis disease activity score; BASDAI: Bath ankylosing spondylitis disease activity index; BASMI 365: Bath ankylosing spondylitis metrology index attained within the last year; RF: rheumatoid factor; antiCCP: cyclic citrullinated peptide antibody; HLA-B27: human leucocyte antibody, subtype B27.

A higher number of women was observed in the higher PDQ classification-groups along with higher absolute values of all patient reported outcomes e.g. patient’s scores for pain, fatigue, global health and disability (HAQ, BASFI), doctor’s global score and disease activity scores for the three diseases (DAS28-CRP, BASDAI, ASDAS). There were no differences across PDQ classification-groups in CRP, serology and current treatment with a biological agent. Furthermore, the median swollen joint count (SJC) was 0 for all categories; however the interquartile range varied slightly (p<0.001).

Some differences across PDQ classification-groups only related to one of the diagnoses:

In patients with RA, higher tender joint count (TJC) and number of patients currently treated with DMARD and previously treated with Biologics were observed in the higher PDQ classification-group, while age was higher in the low PDQ classification-group.

In PsA patients higher absolute values in the higher PDQ-classification-groups for TJC and higher fraction of patients treated with more than one biological drug were seen.

In patients with other SpA a lower proportion of HLA-B27 positive patients was observed in the higher PDQ classification-groups, though not (borderline) statistical significant.

Finally, a stratified analysis of the number of biologic agents received by the patients (first, second, third or fourth or more) did not reveal any differences.

BMI only differed across PDQ-classification-groups for RA-patients (p<0.001), where a higher value was observed in the higher classification-groups, while no difference in smoking habits was observed for any diagnosis (data not shown).

### Swollen to tender joint count ratio

The proportions of STR groups within PDQ classification-group are shown in Table 3. Higher proportions of a low STR, reflecting the single patient having more tender than swollen joints, were seen across PDQ classification-groups with the highest proportions in the high PDQ classification group. A high STR, reflecting the single patient having more swollen than tender joints, was seen in limited proportions of patients across the PDQ classification-groups with the lowest proportion in the high PDQ classification-group. More patients with PsA than with RA had low STR.

**Table 3:** Proportions n (%) of STR groups within PDQ classification-groups.

	Diagnosis	PDQ <13 (n (%))	PDQ 13–18 (n (%))	PDQ >18 (n (%))	p-value
Low STR < 0,5	RA	601(61.4)	310 (60.0)	336 (67.0)	<0.001
	PsA	134 (71.3)	132 (75.4)	149 (78.8)	
Moderate STR 0.5–1.0	RA	317 (32.4)	181 (35.5)	151 (30.0)	0.101
	PsA	48 (25.5)	39 (22.3)	37 (29.6)	
High STR > 1.0	RA	61 (6.2)	26 (5.0)	15 (3.0)	0.045*
	PsA	6 (3.2)	4 (2.3)	3 (1.6)	

Chi square test was used to compare groups. Overall Chi-Square tests: RA, p = 0.03; PsA, p = 0.52.

*Fisher's exact test

### Logistic regression

The results of the logistic regression analysis quantifying the risk of high pain rating are shown in Table 4. The cut off point for the dependent variable ‘VAS pain for PDQ-completers’ was 39 mm. For each of the models, the high and the intermediate, the high and the low, and the intermediate and the low PDQ classification-groups were compared. Also the diagnoses were mutually compared. Of the a priori chosen confounders ‘disease duration’ was insignificant in the adjusted model 1 (p = 0.89) and was therefore omitted in the adjusted model 2. Results were robust across all models. The highest OR’s for having at or above the median VAS pain score were seen in the high PDQ-classification-group compared with the low PDQ classification-group. Thus, it was more likely for the patients in the high PDQ classification group to have a VAS pain score at or above the median than for patients in the intermediate or low PDQ classification-group.

**Table 4:** Logistic regression analysis for modeling high pain status (≥ median VAS pain of 39 mm).

Odds ratio (95%CI)						
	Model 1		Model 1		Model 2	
Variables	unadjusted		adjusted		adjusted	
***PDQgroup H vs*. *I***	2.9	(2.4–3.5)	2.9	(2.3–3.5)	2.9	(2.4–3.5)
***PDQgroup H vs*. *L***	9.6	(8.3–11.6)	10.2	(8.4–12.4)	10.4	(8.6–12.5)
***PDQgroup I vs*. *L***	3.4	(2.9–3.8)	3.6	(3.0–4.2)	3.6	(3.1–4.2)
**PsA vs RA**	1.2	(1.0.-1.4)	1.6	(1.3–1.9)	1.5	(1.3–1.8)
**SpA vs RA**	1.6	(1.4–1.9)	2.3	(1.2–2.3)	2.5	(2.0–3.0)
**SpA vs PsA**	1.4	(1.1–1.6)	1.5	(1.2–1.9)	1.6	(1.3–2.0)

PDQgroup: painDETECT classification-group. H: high (>18), I: intermediate (13–18), L: low (<13).

Prior to regression analysis an analysis of interaction was performed; PDQgroup p<0.001, Diagnosis p< 0.001, PDQgroup*Diagnosis p = 0.76.

Model 1 adjusted: A priori defined potential confounders: sex, age, disease duration, CRP, and Biological treatment (yes/no).

Model 2 adjusted: Omitting ‘disease duration’ (p = 0.89): sex, age, CRP, and Biological treatment (yes/no).

The patients with PsA and SpA were more likely to have a VAS pain score at or above the median compared to patients with RA.

## Discussion

### Pain and pain phenotype prevalence

This registry-based, nationwide cross-sectional study of Danish patients with inflammatory arthritis showed that despite intensive treatment and generally low disease activity scores, 63 percent reported clinical significant residual pain (VAS pain ≥ 30 mm). This number was 52 percent in the overall study population (n = 14339). Clinically acceptable levels of pain in patients with inflammatory diseases are, however, a matter of debate. Wolfe et al., including 12,090 patients with RA, reported the best cut-point for an acceptable level of pain to be 2.0 on a 10 cm VAS [34], whereas Tubach et al., in their study of 1,532 patients with different rheumatologic diagnoses, reported the patient acceptable symptom level to be 42 on a 0–100 scale for pain [35]. It, therefore, seems reasonably to conclude that clinically meaningful pain was present in a large proportion of the study population.

Of the subsample evaluated with the PDQ (n = 7054), more than 20% had a PDQ-score > 18, indicating the presence of primarily neuropathic pain features, while 45–56% depending on diagnosis had a score < 13 indicating the absence of such pain characteristics. In comparison to patients with RA and SpA, patients with PsA more frequently had a PDQ score > 18 (28%) and less frequent a PDQ score < 13 (45%). This observation was further substantiated by higher proportions of a low STR in the high PDQ classification-groups for RA and PsA. Furthermore significantly higher proportions of PsA patients had a low STR, whereas lower proportions had a high STR in comparison to RA.

This also indicated that pain phenotype in PsA patients is harder to distinguish based only on more conventional clinical indices and that other instruments such as the PDQ may be needed.

Overall these findings suggest that contributions from central pain mechanisms may be more prominent in patients with PsA. The existence of a preclinical phase in patients with PsA prior to the diagnosis of the disease has recently been reported [36]. This phase is characterized by nonspecific musculoskeletal symptoms, including joint pain, fatigue, and stiffness preceding the development of PsA; a symptom constellation not unlike the one found in patients with FM. In line, studies report more frequent concomitant FM based on self-report in this patient group [10]. Still, it could be argued that the reporting of more frequent concomitant FM in the PsA group has to do with the nature of the disease involving widespread enthesitis mimicking FM and thus compromising the validity of tools designed to capture FM, including clinical tender point examination. Contradicting this notion is the study by Marchesoni et al reporting tender point to be the most valid clinical discriminator to distinguish PsA from FM [37].

### Characteristics of the different pain phenotypes

Among PDQ-completers significantly and clinically relevant higher levels of pain, fatigue, and negative impact on global health were observed across PDQ classification-groups for all diagnoses with the poorest levels of PROs in the high PDQ classification-group (>18). This tendency was also observed for the TJC and composite disease activity scores (DAS28 and ASDAS), which were found to be low in the low PDQ classification-group and moderate in the intermediate and high PDQ classification-groups. However, no clinically relevant group differences were observed in objective inflammatory indices (SJC, CRP) indicating a larger contribution from non-inflammatory factors to the observed disease activity scores in the high PDQ-group (>18) across all diagnoses.

Doctor’s perception of overall disease activity was strikingly lower than patients self-report and the difference found across pain phenotypes was only borderline clinically relevant reflecting that doctors primarily evaluate the overall disease activity based on more objective indices.

### Association between pain status and PDQ

The logistic regression analysis for the presence of pain at or above the median VAS pain supported that a high PDQ score better explained the presence of high levels of pain than a low score. Thus, patients presenting with neuropathic pain features had a 10-fold higher risk of having high levels of pain.

### Clinical implications

In the absence of any diagnostic gold standards, mechanism-based pain classification is based on the assumption that this can be done clinically, based on identifiable and discriminatory patterns of symptoms and signs assumed to reflect the underlying pathophysiology. Current scientific evidence, however, suggests that both neuropathic pain and pain conditions characterised primarily by augmented central pain processing may share similar neurobiological underpinnings [38]. Given that these neurobiological underpinnings give rise to clinical symptoms, it is not surprising if centrally mediated pain and neuropathic pain may share common clinical features such as those captured by the PDQ. Substantiating this notion, striking phenotypic similarities has been demonstrated in patients with established peripheral neuropathy and FM when evaluated with the PDQ [16]. It could, therefore, be speculated that rather than indicating the presence of neuropathic pain, a high PDQ score in our patient population reflected the presence of a primarily central pain component, including concomitant fibromyalgia. It is well recognized that compared to individuals with localized or regional pain, patients with centralized pain syndromes, including FM, report a higher illness burden; higher pain intensity, more pronounced pain-related interference with everyday life, and higher levels of psychological distress [39]. In the original study by Freynhagen et al., introducing the PDQ to an unselected sample of 8000 patients with chronic low back pain, 37% were classified as having a primarily neuropathic pain component [12]. Also in this sample, a high PDQ score was found to be associated with more intense pain, more severe co-morbidity and poorer quality of life. From a critical point of view, it could be argued that a high score on the PDQ might simply be related to the tendency to report more symptoms in general, which might also be linked to psychosocial factors, or represent a non-specific finding in patients with more centralized pain syndromes.

Still, since pain is frequently used as a proxy for inflammation in the evaluation of rheumatic diseases, it is important to recognize that not all pain is inflammatory. Interestingly, the study indicated a more intensive level of treatment across PDQ classification-groups. Although there were no differences in the numbers of patients receiving biological treatment across PDQ classification-groups, patients in the high PDQ classification-group were more likely to have received more than one biological agent (PsA) and to have been changed back to DMARDs after having been treated with biological agents (RA). These findings further indicate that treatment failure is more prevailing in patients with signs of augmented central pain processing [40]. Previous reports of worse treatment outcome for RA and PsA patients with concomitant fibromyalgia support this observation [5;41].

It has been proposed that disease duration is related to the development of pain hypersensitivity in RA [42]. The results of this study did not support this notion as no significant differences were found across the PDQ classification-groups. In contrast, a statistically significant predominance of female gender was observed in the high PDQ group across all diagnoses, indicating a higher frequency of central pain mechanisms among females. This finding is in accordance with the observed gender distribution in other pain conditions characterized by augmented central pain processing, for example, FM [43;44].

### Limitations

The study had some limitations: the data sampled from the DANBIO registry were not complete; however, considering the large sample size we find that the descriptive statistics were representative. The more than 7,000 patients accepting to participate in the PDQ-survey had higher ratings on VAS pain and other PRO’s than those declining, which might have skewed the distribution of PDQ-scores. Detailed general demographic and clinical information, including information about possible comorbidities associated with neuropathic pain, were not obtainable in this registry-based study and may also have influenced the study findings. The study design being cross-sectional did not allow for advanced multivariable modelling, which could have added more unique information on association between certain variables or characteristics and pain phenotype. More prospective studies involving prognostic factor research are desirable.

Furthermore, although the PDQ has been used to assess neuropathic pain features as a proxy of central sensitization, the cut off values were established and validated in a sample of neuropathic pain patients with a clearly defined peripheral factor, e.g. polyneuropathy and post herpetic neuralgia. The traditional PDQ cut off values may therefore not be valid for the concept of central sensitization in patients with inflammatory disorders. However, aside from construct validity, our group has shown that the PDQ has acceptable psychometric properties, including unidimensionality, scaling properties and test-retest reliability when applied in patients with inflammatory arthritis (in review). Lastly, this registry-based study did not examine if patients also fulfilled the criteria for a diagnosis of FM; a pain condition that by many is considered to represent the upper end of a pain hypersensitivity continuum. However, the existence of a gradual transition from acute physiological pain to more chronic pain states driven by changes in neural processing in the central nervous system is increasingly being recognized [2]. Nociceptive pain and pain caused by central pain mechanisms should therefore not be regarded as exclusive categorical labels but rather as concurrent possible mechanistic contributors to the patient’s pain. Because of the likely influence of pain hypersensitivity on clinically relevant outcomes, the entire clinical pain hypersensitivity spectrum among patients with inflammatory disorders should be captured. Instruments designed to assess underlying pain mechanisms, and not only symptom based instruments designed to diagnose FM, therefore seem to be of relevance for this patient population.

### Conclusion

In conclusion, more than 50% of this large Danish population of patients with inflammatory arthritis reported significant pain and among those who completed the PDQ, approximately 20% were classified as having primarily neuropathic pain features; a possible proxy for central sensitization. The PDQ-score was associated with composite disease activity scores and PROs, but not with “objective markers” of peripheral inflammation (CRP and swollen joint count). Patients with indication of a central pain mechanism were found to have a 10-fold higher risk of having a high pain level than those with an indication of non-neuropathic pain. Pain classification by PDQ in patients with inflammatory arthritis may assist the advancement of pain mechanism-based treatment approaches and alignment of the expected outcome of anti-inflammatory treatment.

### Ethical considerations

Patient consent was obtained on the touch screen prior to the redirection to the PDQ. According to Danish legislation, surveys do not require approval by Ethics Committees and registrations and publications of data from clinical registries do not require patient consent or approval by Ethics Committees. Approval was obtained from the Danish Data Protection Agency.
